# Mediating role of body composition and insulin resistance on the association of arterial stiffness with blood pressure among adolescents: The ALSPAC study

**DOI:** 10.3389/fcvm.2022.939125

**Published:** 2022-09-02

**Authors:** Andrew O. Agbaje

**Affiliations:** Faculty of Health Sciences, School of Medicine, Institute of Public Health and Clinical Nutrition, University of Eastern Finland, Kuopio, Finland

**Keywords:** obesity, hypertension, pediatrics, health promotion, mediation, epidemiology, early vascular aging, atherosclerosis

## Abstract

**Background:**

Emerging evidence among adolescents suggests that arterial stiffness temporally precedes elevated blood pressure/hypertension in the casual pathway. It remains unknown whether insulin resistance and body composition mediate this relationship. Therefore, we examined the mediating role of total fat mass, lean mass, and insulin resistance in the association between arterial stiffness and blood pressure among adolescents.

**Materials and methods:**

We studied 3,764 participants, aged 17 years from the Avon Longitudinal Study of Parents and Children (ALSPAC) United Kingdom birth cohort. Arterial stiffness accessed with Vicorder device measured carotid-femoral pulse wave velocity (cfPWV), body composition was measured by dual-energy Xray Absorptiometry, blood pressure by Omron device, and homeostatic model assessment of insulin resistance (HOMA-IR) was computed. Data were analysed with structural equation models mediation path analyses and adjusted for cardiometabolic and lifestyle factors.

**Results:**

Among 1,678 [44.6%] male and 2,086 [55.4%] female participants, higher cfPWV was directly and independently associated with higher systolic and diastolic blood pressure, irrespective of the mediator [Standardized regression coefficient (β) = 0.248–0.370, *p* for all = 0.002]. Lean mass [β = 0.010; *p* = 0.026; 3.3% mediation] and HOMA-IR [β = 0.004; *p* = 0.033; 1.1% mediation] but not total fat mass [β < 0.0001; *p* = 0.615; 0% mediation] partly mediated the association of cfPWV with systolic blood pressure after full adjustments. Similarly, lean mass [β = –0.004; *p* = 0.021; 1.4% mediation] and HOMA-IR [β = 0.007; *p* = 0.039; 2.8% mediation] but not total fat mass [β = –0.002; *p* = 0.665; 0.7% mediation] partly mediated the association of cfPWV with diastolic blood pressure.

**Conclusion:**

Attenuating insulin resistance may be a potentially valuable strategy in lowering higher blood pressure precipitated by higher arterial stiffness.

## Introduction

The prevalence of elevated blood pressure and hypertension in youth remains high despite interventions targeted at decreasing the global burden (1–4). It has been established among adults that elevated blood pressure and hypertension may be caused by arterial stiffness (5, 6). Recently, this evidence was confirmed in a prospective study conducted among adolescents and young adults (1). In addition, arterial stiffness appears to temporally precede insulin resistance whereas higher arterial stiffness bi-directionally associate with higher fat mass and lean mass (1, 7).

The prevention of elevated blood pressure and hypertension requires further understanding of the mechanistic path through which causative factors such as arterial stiffness exert their influence (1, 4, 6–9). Clinical trials to lower arterial stiffness in order to decrease elevated blood pressure and hypertension have recorded limited success, likely due to the small sample size (6, 10). Since arterial stiffness causally associate with insulin resistance, fat mass, lean mass, and elevated pressure, it remains unknown whether insulin resistance and body composition mediate the effect of arterial stiffness on elevated blood pressure in youth. Therefore, we examined the mediating role of body composition and insulin resistance on the association of arterial stiffness with blood pressure among adolescents using data from the ALSPAC (Avon Longitudinal Study of Parents and Children) birth cohort, England, United Kingdom.

## Materials and methods

### Study cohort

Data were from the ALSPAC birth cohort, which investigates factors that influence childhood development and growth. Altogether, 14,541 pregnancies from women residing in Avon, southwestern England, United Kingdom, who had a total of 14,676 fetuses, were enrolled between April 1, 1991, and December 31, 1992. When the oldest children were approximately 7 years of age, an attempt was made to bolster the initial sample with eligible cases who had failed to join the study originally resulting in 913 additional pregnancies. The total sample size for analyses using any data collected after 7 years of age is 15,454 pregnancies, resulting in 15,589 fetuses. Of these 14,901 were alive at 1 year of age. Regular clinic visits of the children commenced at 7 years of age and are still ongoing. For our analysis, we included participants who had complete fat mass, lean mass, blood pressure, and carotid-femoral pulse wave velocity (cfPWV) measurements at the age 17-year clinic visit. The demographic characteristics of excluded participants were similar to those included in this study as reported earlier (1). Ethical approval for the study was obtained from the ALSPAC Ethics and Law Committee and the Local Research Ethics Committees. Informed consent for the use of data collected *via* questionnaires and clinics was obtained from participants following the recommendations of the ALSPAC Ethics and Law Committee at the time (11–13). Consent for biological samples has been collected in accordance with the Human Tissue Act (2004).

### Anthropometry and body composition

Anthropometry (height and weight) at 17 years of age was assessed using a stadiometer (SECA 213, Birmingham, United Kingdom) and body mass (kilogram) using electronic weighing scales (Marsden M-110, Rotherham, United Kingdom) (1, 7). Body composition (total fat mass and lean mass) was assessed using a dual-energy Xray absorptiometry (GE Medical Systems, Madison, WI, United States) scanner as earlier described (1, 7). We calculated body mass index by dividing weight by squared height.

### Vascular phenotype

At age 17-year clinic visit, cfPWV arterial measure was recorded three times. A cuff was placed over the right carotid artery in the participant’s neck, while another was located over the femoral artery in their upper right thigh. The distance between the participant’s suprasternal notch and the top of the thigh cuff was measured, as was the distance between their suprasternal notch and the bottom of the neck cuff on the right side. cfPWV and transit time to the nearest 0.01 ms were automatically computed from measurements of pulse transit time and distance traveled by the pulse between two recording sites using Vicorder (Skidmore Medical, Bristol, United Kingdom) portable physiologic vascular testing equipment. All measurements were taken independently by one of two trained vascular technicians (inter-observer mean difference 0⋅2 m/s, SD 0⋅1) (1, 7).

### Cardiometabolic and lifestyle factors

Heart rate, systolic and diastolic blood pressure were measured at 17 years of age. Blood pressure was measured twice while the participants were at rest using the appropriate cuff size for the upper arm circumference, and the mean of each was recorded. Blood pressure was measured in the seated position using an Omron 705-IT at the 17-years of age clinic visit (1, 7). Using standard protocols, fasting blood samples at ages 17 years were collected, spun, and frozen at –80°C, and a detailed assessment of glucose, insulin, high sensitivity C-reactive protein, low-density lipoprotein cholesterol, high-density lipoprotein cholesterol, and triglycerides, has been reported (coefficient of variation was < 5%) (1, 7). Specifically, fasting insulin was measured using an ultrasensitive automated microparticle enzyme immunoassay (Mercodia), which does not cross-react with proinsulin. The sensitivity of the immunoassay was 0.07 mU/L. We calculated the homeostatic model assessment of insulin resistance (HOMA-IR) from (fasting plasma insulin x fasting plasma glucose/22.5) (14).

A questionnaire to assess smoking behavior was administered at the 17-year clinic visit. The participants were asked whether they smoked in the last 30 days, smoked a whole cigarette, smoked every day, their frequency of use, etc. Moreover, participants were briefly asked about their personal and family (mother, father, and siblings) medical history such as a history of hypertension, diabetes, high cholesterol, and vascular disease. Moderate to vigorous physical activity at ages 15.5 years was assessed with ActiGraphTM accelerometer worn for 7 days. Moderate to vigorous physical activity cut point was > 2,296 counts per minute (15).

### Statistical analysis

Participants’ descriptive characteristics were summarized as means and standard deviation, medians and interquartile ranges, or frequencies and percentages. We explored sex differences using Independent *t*-tests, Mann Whitney-U tests, or Chi-square tests for normally distributed, skewed or dichotomous variables, respectively. We assessed the normality of variables by histogram curve. We conducted a logarithmic transformation of skewed variables and confirmed normality prior to further analysis.

We used structural equation model mediating path analyses to examine the separate mediating role of total fat mass, lean mass, and insulin resistance in the associations of cfPWV, with each systolic and diastolic blood pressure. Analyses were adjusted for age, sex, high-density lipoprotein cholesterol, low-density lipoprotein cholesterol, triglyceride, high-sensitivity C-reactive protein, family history of hypertension and cardiovascular diseases, smoking status, heart rate, and insulin, total fat mass or lean mass depending on the mediator. The path models had three equations per regression analysis: the association of cfPWV with total fat mass, lean mass, or HOMA-IR (Equation 1); the association of total fat mass, lean mass, or HOMA-IR with systolic and diastolic blood pressure (Equation 2); and the association of cfPWV with systolic and diastolic blood pressure (Equation 3), and Equation 3’ accounted for the mediating role of fat mass, lean mass or HOMA-IR. The proportion of mediating roles was estimated as the ratio of the difference between Equation 3 and Equation 3’ or the multiplication of Equations 1 and 2 divided by Equation 3 and expressed in percentage. A mediating or indirect role is confirmed when there are statistically significant associations between (a) the predictor and mediator, (b) the predictor and outcome, (c) the mediator and outcome, and when (d) the association between the predictor and outcome variable was attenuated upon inclusion of the mediator (16, 17). Path analyses were conducted with 1,000 bootstrapped samples.

All covariates were selected based on previous studies (1, 4, 7, 17, 18). We excluded pubertal status/somatic maturation from the model because all participants had reached adult-like maturity status by 17 years of age. We considered differences and associations with a 2-sided *p*-value < 0.05 as statistically significant and made conclusions based on standardized regression coefficients and *p*-value. Missing data were accounted for using regression imputations within the structural equation model prior to mediation analysis. Studies involving four thousand ALSPAC children with 0.8 statistical power, 0.05 alpha, and 2-sided *p*-value would show a minimum detectable effect size of 0.049 standard deviations if they had relevant exposure to a normally distributed quantitative variable (19). All descriptive statistical analyses were performed using SPSS statistics software, Version 27.0 (IBM Corp, Armonk, NY, United States), and structural equation modeling mediating path analyses were conducted using IBM AMOS version 27.0.

## Results

### Cohort study characteristics

In the ALSPAC birth cohort, 14,901 children were alive at 1 year of age, of whom 5,217 adolescents participated in the follow-up clinic visit. Altogether 3,764 participants who had complete total fat mass, lean mass, blood pressure, and cfPWV measurements at 17 years of age were included in the present study. Females had a lower height, weight, lean mass, systolic blood pressure, and cfPWV but higher body mass index and total fat mass than males. Other participants’ characteristics are shown in Table 1.

**TABLE 1:** Descriptive characteristics of cohort participants.

Variables	Male		Female		*P*-value
			
	*N*	Mean (SD)	*N*	Mean (SD)	
**Anthropometry**					
Age (years)	1,678	17.72 (0.32)	2,086	17.72 (0.34)	0.876
Height (m)	1,667	1.79 (0.07)	2,078	1.65 (0.06)	<0.0001
*Weight (kg)	1,670	70 (14.7)	2,080	60.3 (13.3)	<0.0001
Race- White (n,%)	1,524	1,457 (95.6)	1,879	1,803 (95.9)	0.608
**Body composition**					
*Total fat mass (kg)	1,678	10.32 (9.9)	2,086	19.1 (10.12)	<0.0001
*Lean mass (kg)	1,678	54.97 (8.2)	2,086	37.94 (5.27)	<0.0001
*Body mass index (kg/m^2^)	1,667	21.6 (4.08)	2,078	22.02 (4.32)	0.001
**Metabolic profile**					
High density lipoprotein (mmol/L)	1,247	1.18 (0.26)	1,280	1.35 (0.31)	<0.0001
Low density lipoprotein (mmol/L)	1,247	2.00 (0.56)	1,280	2.21 (0.63)	<0.0001
*Triglyceride (mmol/L)	1,247	0.74 (0.35)	1,280	0.77 (0.37)	0.082
Glucose (mmol/L)	1,247	5.16 (0.70)	1,280	4.91 (0.50)	<0.0001
*Insulin (mU/L)	1,229	6.08 (4.26)	1,255	7.33 (4.48)	<0.0001
*HOMA-IR	1,220	1.38 (1.04)	1,252	1.61 (1.01)	<0.0001
*High sensitivity C-reactive protein (mg/L)	1,247	0.44 (0.69)	1,280	0.67 (1.30)	<0.0001
**Vascular measures**					
Heart rate (beats per minute)	1,678	63 (9)	2,086	67 (10)	<0.0001
Systolic blood pressure (mmHg)	1,678	120 (9)	2,086	110 (8)	<0.0001
Diastolic blood pressure (mmHg)	1,678	63 (6)	2,086	65 (6)	<0.0001
*Carotid-femoral pulse wave velocity (m/s)	1,678	5.98 (0.84)	2,086	5.47 (0.75)	<0.0001
**Lifestyle factors**					
Smoked cigarettes (*n*, %)	1,462	362 (24.8)	1,812	521 (28.8)	0.011
MVPA (mins/day)	678	56 (31)	866	40 (23)	<0.0001
Family history of H-D-C-V (*n*, %)	1,677	485 (28.9)	2,083	653 (31.4)	0.108

The values are means (standard deviations) and *median (interquartile range) except for categorical variables in frequency and percentage. Differences between sexes were tested using Student’s *t*-test for normally distributed continuous variables, Mann–Whitney U test for skewed continuous variables, and Chi-square test for dichotomous variable. A 2-sided *P*-value < 0.05 is considered statistically significant. H-D-C-V, hypertension/diabetes/high cholesterol/vascular disease; MVPA, moderate to vigorous physical activity; NA, not available/applicable; BP, blood pressure. *p*-value for sex difference.

### Mediating role of total fat mass, lean mass, and insulin resistance on the associations of carotid-femoral pulse wave velocity with systolic and diastolic blood pressure

**FIGURE 1:**
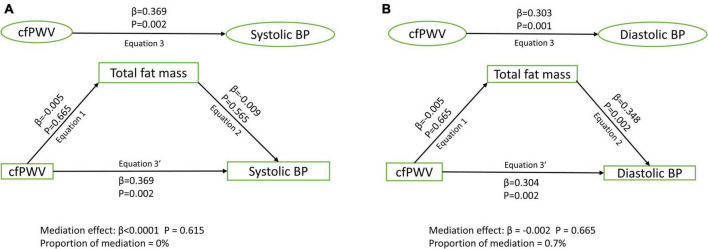
Mediating role of fat mass on the associations of arterial stiffness with systolic blood pressure **(A)** and diastolic blood pressure **(B)**. Mediating path analyses were adjusted for age, sex, high-density lipoprotein cholesterol, low-density lipoprotein cholesterol, triglyceride, high-sensitivity C reactive protein, family history of hypertension and cardiovascular diseases, smoking status, heart rate, insulin, and lean mass. BP, blood pressure; cfPWV, carotid-femoral pulse wave velocity.

**FIGURE 2:**
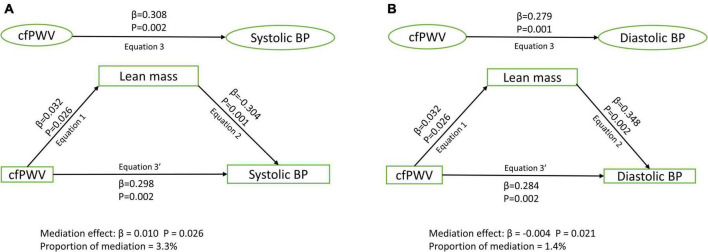
Mediating role of lean mass on the associations of arterial stiffness with systolic blood pressure **(A)** and diastolic blood pressure **(B)**. Mediating path analyses were adjusted for age, sex, high-density lipoprotein cholesterol, low-density lipoprotein cholesterol, triglyceride, high-sensitivity C reactive protein, family history of hypertension and cardiovascular diseases, smoking status, heart rate, insulin, and fat mass. BP, blood pressure; cfPWV, carotid-femoral pulse wave velocity.

**FIGURE 3:**
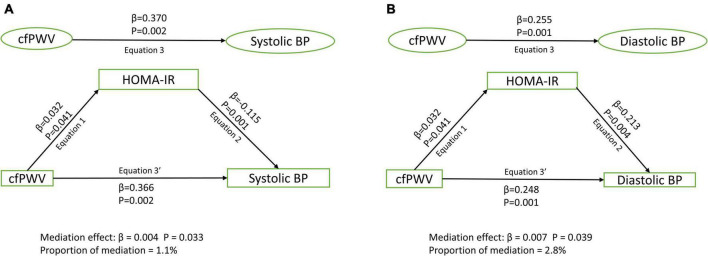
Mediating role of insulin resistance on the associations of arterial stiffness with systolic blood pressure **(A)** and diastolic blood pressure **(B)**. Mediating path analyses were adjusted for age, sex, high-density lipoprotein cholesterol, low-density lipoprotein cholesterol, triglyceride, high-sensitivity C reactive protein, family history of hypertension and cardiovascular diseases, smoking status, heart rate, fat mass, and lean mass. BP, blood pressure; cfPWV, carotid-femoral pulse wave velocity; HOMA-IR, homeostatic model assessment of insulin resistance.

After adjusting for cardiometabolic and lifestyle factors, total fat mass had no mediating role in the associations of arterial stiffness with systolic and diastolic blood pressure (Figures 1A,B). Lean mass mildly mediated the associations of arterial stiffness with systolic blood pressure and diastolic blood pressure (Figures 2A,B). Insulin resistance had a mild mediating effect on the relationships of arterial stiffness with systolic and diastolic blood pressure (Figures 3A,B).

## Discussion

In a large cohort of apparently healthy adolescents, we present novel findings on the mediating role of body composition and insulin resistance in the associations of arterial stiffness with systolic and diastolic blood pressure. We observed that insulin resistance and lean mass but not fat mass mildly moderated the relationship between higher arterial stiffness and higher blood pressure. These findings suggest that arterial stiffness may influence blood pressure *via* a higher insulin resistance pathway, rather than an increased fat mass path.

Recent evidence among adolescents and previous reports among adults concluded that arterial stiffness may temporally cause elevated blood pressure and hypertension (1, 5, 6, 8). Interventions to reverse or prevent arterial stiffness in order to lower blood pressure have been limited (10), necessitating further studies on ways to understand the pathological influence of arterial stiffness. The role of obesity or higher fat mass in increasing blood pressure and arterial stiffness has been well-documented in the pediatric population (20–24). However, we observe that arterial stiffness may not exact its influence on blood pressure through a fat mass pathway. Arterial stiffness is bi-directionally associated with fat mass which means that higher fat mass predicts higher arterial stiffness (1, 22) and *vice versa* (1). It is plausible that higher fat mass increases the likelihood of higher arterial stiffness which in turn directly increases blood pressure (6, 25). Nonetheless, an increase in blood pressure mainly due to a higher arterial stiffness may not be reversed simply by reducing fat mass, particularly among adolescents who are mainly normal weight. Our findings may differ among adolescents who are overweight and obese where higher fat mass may predispose to higher arterial stiffness and subsequently higher blood pressure (25, 26). Controlling for lipids, smoking status, and other cardiometabolic and lifestyle covariates might have dampened the mediating role of fat mass in the relationship between arterial stiffness and blood pressure. These covariates were independently associated with both arterial stiffness and blood pressure in adolescents (6, 25, 26). Further studies are warranted to investigate whether the possible effect of fat mass in the relationship between arterial stiffness and blood pressure could exist *via* a lipid mediating pathway.

Lean mass appears to have a mild mediating role in the association of arterial stiffness and blood pressure. Higher lean mass has been associated with higher arterial stiffness and blood pressure in adolescents (1, 27, 28). Higher lean mass mediating role may be related to an increase in stroke volume and cardiac output in response to cardiovascular system adaptation (1, 27). Higher arterial stiffness may also trigger increased angiogenesis which may lead to increased muscle growth and higher blood volume, thus raising the blood pressure in adolescents (1). However, among adults lower lean or muscle mass has been associated with higher arterial stiffness (29). Taken together, higher arterial stiffness may lead to higher lean mass which in turn may result in higher blood pressure among healthy adolescents, reflecting a physiologic rather than a pathologic process. Our findings suggest that higher arterial stiffness may elicit an indirect physiologic response *via* increased lean mass resulting in elevated blood pressure.

We observed that insulin resistance partly mediated the relationship between higher arterial stiffness and increased blood pressure. Recently, it was shown that arterial stiffness in adolescence may be a precursor of insulin resistance in young adulthood (7). Similarly, the present mediation analysis revealed that higher arterial stiffness as a predictor was associated with higher insulin resistance as an outcome among adolescents. We also found that higher insulin resistance was associated with higher systolic and diastolic blood pressure independent of fat mass, cardiometabolic, and lifestyle factors. The relationship between higher insulin resistance and higher blood pressure independent of obesity has been reported earlier among adolescents (30). Several mechanisms have been proposed linking insulin resistance to elevated blood pressure such as excess circulating insulin increasing renal sodium absorption (31), increased sympathetic nervous system activity (32), and vascular smooth muscle proliferation (33). However, our findings suggest a plausible additional mechanism where higher arterial stiffness leads to higher insulin resistance which later culminates in increased blood pressure. Arterial stiffness attenuates and impedes blood flow to high-flow, low-resistance organs such as the liver and pancreas, which creates a cascade of events such as insulin resistance, hyperinsulinemia, and subsequently elevated blood pressure (6, 7, 34). This arterial stiffness-insulin resistance-blood pressure path may explain approximately three percent of the associations between arterial stiffness and blood pressure, especially in healthy adolescents. The mediating role of insulin resistance may likely be much higher among adolescents at high cardiometabolic risks, but further studies are warranted.

The strengths of this study include the use of dual-energy Xray absorptiometry-derived body composition measures, in contrast to body mass index adiposity measure. Extensively phenotyped birth cohort with several objective assessments of cardiometabolic and lifestyle factors. A gold-standard measure of arterial stiffness (6, 34) and a very large adolescent population who were apparently healthy. The application of advanced statistical tools, such as mediation path analysis offers some causal explanation but the quality of the evidence would have been higher if the analysis were prospective rather than cross-sectional. Nonetheless, clinical trials are the gold standard for inferring causality. Other limitations of our study include few participants with diagnosed diseases which underpower any analysis examining the hypothesis in a diseased sub-group. Our participants were mostly Caucasian; thus, our findings may not be generalizable to other racial groups. We could not exclude the possibility of residual confounding of other unmeasured variables, such as dietary factors, but metabolic indices are often correlated with the dietary pattern. The blood pressure assessment may vary depending on the time of the day it was collected. We could not control for the specific time in the day when blood pressure was assessed because the data was not available, but error terms were included in the analyses to account for variability.

## Conclusion

In adolescents, total fat mass did not mediate the association of arterial stiffness with increasing systolic and diastolic blood pressure. Lean mass and insulin resistance had a mild mediating role in the association of higher arterial stiffness with higher blood pressure. Therefore, attenuating insulin resistance may be a potentially valuable strategy in lowering higher blood pressure precipitated by higher arterial stiffness. Intervention studies targeted at lowering insulin resistance are warranted in the adolescent population.

## Data availability statement

The datasets presented in this article are not readily available because the informed consent obtained from ALSPAC participants does not allow the data to be made freely available through any third-party maintained public repository. However, data used for this submission can be made available on request to the ALSPAC Executive. The ALSPAC data management plan describes in detail the policy regarding data sharing, which is through a system of managed open access. Full instructions for applying for data access can be found here: http://www.bristol.ac.uk/alspac/researchers/access/. The ALSPAC study website contains details of all the data that are available (http://www.bristol.ac.uk/alspac/researchers/our-data/).

## Ethics statement

The studies involving human participants were reviewed and approved by Avon Longitudinal Study of Parents and Children Ethics and Law Committee and the Local Research Ethics Committees, University of Bristol, United Kingdom. Written informed consent to participate in this study was provided by the participants’ legal guardian/next of kin.

## Author contributions

AA had full access to all the data in the study, took responsibility for the integrity of the data and the accuracy of the data analysis, did concept and design, acquisition, analysis, or interpretation of data, drafting of the manuscript, critical revision of the manuscript for important intellectual content, statistical analysis, obtained funding, and approved the submitted version.
